# Trajectories of the Framingham general cardiovascular risk profile in midlife and poor motor function later in life: The Whitehall II study^☆^^☆☆^

**DOI:** 10.1016/j.ijcard.2013.12.051

**Published:** 2014-03-01

**Authors:** Alexis Elbaz, Martin J. Shipley, Hermann Nabi, Eric J. Brunner, Mika Kivimaki, Archana Singh-Manoux

**Affiliations:** a INSERM, Centre for Research in Epidemiology and Population Health, U1018, Social and Occupational Determinants of Health, F-94807, Villejuif, France; b Department of Epidemiology and Public Health, University College London, UK; c Univ Versailles St-Quentin, UMRS 1018, F-94807, Villejuif, France; d Centre de Gérontologie, Hôpital Ste Périne, AP-HP, France

**Keywords:** CVD, cardiovascular disease, FRS, Framingham general cardiovascular disease risk score, SES, socioeconomic status, BMI, body mass index, SD, standard deviation, Cardiovascular risk score, Motor function, Aging, Stroke, Cohort study

## Abstract

**Background:**

Vascular risk factors are associated with increased risk of cognitive impairment and dementia, but their association with motor function, another key feature of aging, has received little research attention. We examined the association between trajectories of the Framingham general cardiovascular disease risk score (FRS) over midlife and motor function later in life.

**Methods:**

A total of 5376 participants of the Whitehall II cohort study (29% women) who had up to four repeat measures of FRS between 1991–1993 (mean age = 48.6 years) and 2007–2009 (mean age = 65.4 years) and without history of stroke or coronary heart disease in 2007–2009 were included. Motor function was assessed in 2007–2009 through objective tests (walking speed, chair rises, balance, finger tapping, grip strength). We used age- and sex-adjusted linear mixed models.

**Results:**

Participants with poorer performances for walking speed, chair rises, and balance in 2007–2009 had higher FRS concurrently and also in 1991–1993, on average 16 years earlier. These associations were robust to adjustment for cognition, socio-economic status, height, and BMI, and not explained by incident mobility limitation prior to motor assessment. No association was found with finger tapping and grip strength.

**Conclusions:**

Cardiovascular risk early in midlife is associated with poor motor performances later in life. Vascular risk factors play an important and under-recognized role in motor function, independently of their impact on cognition, and suggest that better control of vascular risk factors in midlife may prevent physical impairment and disability in the elderly.

## 1 Introduction

In addition to being strong predictors of cardio- and cerebrovascular disease, vascular risk factors have been associated with aging phenotypes, including worse cognitive function [1] and dementia [2]. Motor impairment is another key aspect of the aging process and poor motor function has been linked to adverse health outcomes, including disability [3] and death [4]. However, the association of vascular risk factors with motor function has received little research attention. To date, there is some evidence linking individual risk factors (hypertension [5], diabetes [6]) or markers of subclinical atherosclerosis [7–9] to poorer motor function, but the combined effect of vascular risk factors remains unknown.

For better prediction of cardiovascular disease (CVD) risk and a more complete assessment of vascular burden, several risk algorithms encompassing multiple risk factors have been developed [10]. Scores derived from these algorithms predict the risk of CVD, stroke, dementia [11], and cognitive deficit [12,13], but to our knowledge their association with motor function has not been examined. Here, we examine the association between trajectories of the Framingham general cardiovascular disease risk score (FRS) [14] during midlife, using four assessments over 16 years, and motor function at the end of the follow-up.

## 2 Methods

### 2.1 Participants

The Whitehall II study is a longitudinal study of 10,308 civil servants [15]. All civil servants aged 35–55 years in 20 London based departments were invited to participate (participation rate, 73%). The baseline examination took place over 1985–1988. Subsequent phases including clinical examinations and questionnaires were in 1991–1993, 1997–1999, 2002–2004, and 2007–2009. Participants gave written consent; the University College London ethics committee approved the study.

### 2.2 Motor function

Motor function was assessed in 2007–2009 through measures of walking speed, chair rises, balance, grip strength, and finger tapping; while the first three tests involve several systems and represent global measures, the last two are taken at the upper limbs and represent more specific measures (muscle strength, psychomotor speed). A practice session was allowed for all. Correlations between tests were weak to moderate (supplementary Table 1).

*Walking speed* was measured at usual pace over a marked 8-ft (2.44 m) course. The starting position was standing at the start of the course. A trained nurse walked behind the participant and stopped timing when the participant's foot hit the floor after the end of the course. Three tests were conducted; walking speed was computed as 2.44 m divided by the mean of three measures (in seconds).

*Time to complete 5 chair rises*: participants sat on an armless chair with feet resting on the floor and arms folded across their chest. They stood up without using their arms and sat down five times as quickly as possible. Time needed to complete the five chair rises was recorded. Participants (n = 6) not able to stand up five times were excluded.

*Balance* was assessed through a series of tests of varying difficulty (full- and semi-tandem stands, one-leg balance with eyes open or closed). For the present analyses, we used data from the full-tandem stand and one-leg balance test with eyes open. Participants were first asked to perform a full-tandem stand (10 s). If they passed this test, they proceeded to perform a one-leg balance test (30 s). Participants who failed either test were deemed to have failed the balance test.

*Grip strength* (in kilograms; dominant hand) was measured using a Smedley hand grip dynamometer adjusted to suit participants' hands with participants seated, their elbow on the table, forearm pointing upwards, and palm of the hand facing up. Participants were asked to squeeze the dynamometer as hard as possible for 2 s. Three tests were performed with a one minute rest between each. Readings were rounded up to the nearest whole number; the mean of the tests was used.

*Finger tapping test*: the number of taps during 10 s was recorded using an electronic device (WPS Electronic tapping test) [16] with participants holding their dominant hand palm down, fingers extended, keeping their hand and arm stationary, and tapping on the lever using their index.

### 2.3 Framingham general cardiovascular disease risk score

The FRS was developed as part of the Framingham Heart study to assess general CVD risk and risk of individual events (coronary, cerebrovascular, peripheral artery disease, heart failure) [14]. It includes measures of age, HDL- and total cholesterol, systolic blood pressure, cigarette smoking, and diabetes, and provides an estimate of the 10-year risk of CVD.

Risk score components were drawn from questionnaires and clinical examination data at four waves: 1991–1993, 1997–1999, 2002–2004, and 2007–2009. Total cholesterol and HDL cholesterol (mg/dL) were measured from blood collected after either an 8 h fast (participants presenting in the morning), or at least 4 h after a light fat-free breakfast (participants presenting in the afternoon). Cholesterol was measured using a Cobas Fara centrifugal analyzer (Roche Diagnostics System). HDL cholesterol was measured by precipitating non-HDL cholesterol with dextran sulfate-magnesium chloride and measuring cholesterol in the supernatant fluid. Systolic blood pressure (mm Hg) was taken as the average of two measurements in the sitting position after a 5 min rest with the Hawksley random-zero sphygmomanometer. Treated hypertension was determined according to antihypertensive medication use. Participants were categorized as current smokers, ex- or non-smokers. Diabetes was defined by fasting glucose ≥ 7.0 mmol/L, 2 h post-load glucose ≥ 11.1 mmol/L, doctor diagnosed diabetes, or use of diabetes medication [17].

### 2.4 Covariates

Individuals with prevalent or incident stroke or coronary heart disease (non-fatal myocardial infarction, definite angina) between 1991–1993 and 2007–2009 were excluded as these conditions are known to affect motor performances. Myocardial infarction was diagnosed based on clinical examination data, electrocardiograms, and medical records [18]. Angina was assessed based on reports of symptoms and nitrate medication, with corroboration in medical records or abnormalities on a resting electrocardiogram, exercise electrocardiogram, or coronary angiogram. Classification was carried out independently by two trained coders, with adjudication by a third party in the event of disagreement. Stroke was self-reported and included history of stroke or transient ischemic attack.

At all waves, mobility limitations were assessed using questions on the ability to climb several flights of stairs or walk more than 1 mile. Socioeconomic status (SES) was defined based on the highest 3-level British civil service employment grade achieved (high, administrative; intermediate, professional or executive; low, clerical or support). Weight and height were measured and body mass index (BMI) was calculated as weight divided by height squared (kg/m^2^). Cognitive status was assessed using the Alice Heim 4-I (AH4-I) test [19], which includes 65 verbal and mathematical reasoning items assessing inductive reasoning by measuring the ability to identify patterns and infer principles and rules; higher scores correspond to better function. Participants had 10 min to do this test.

### 2.5 Statistical analysis

Descriptive analyses were carried out to examine participants' characteristics at each wave and their association with motor tests and FRS in 2007–2009. Correlations between z-scores of motor tests were examined through age- and sex-adjusted partial Spearman correlations.

The association between the FRS and motor tests was examined separately for each test to establish whether motor function was associated with FRS concurrently and with FRS trajectories over 16 years prior to motor testing. We defined age- and sex-specific quartiles (supplementary Table 2) for all tests with continuous measures (walking speed, grip strength, finger tapping, chair rises), given that motor performances decreased with age (p < 10^− 4^) and were higher in men than women (p < 10^− 4^) [20]. For balance (binary measure), models were age- and sex-adjusted.

We used linear mixed models that take into account correlations between repeated measures on the same individual, with FRS as the dependent variable and quartiles of motor tests as independent variables; this approach allows examining FRS trajectories prior to the measurement of motor function (2007–2009) as well as their concurrent association. FRS was logarithmically transformed due to its skewed distribution; results were back-transformed for graphs. Models were implemented with a backward time scale, so that 2007–2009 corresponds to the baseline (time = 0) and participants are tracked back until 1991–1993, approximately 16 years earlier. Time was divided by 10, so that regression coefficients represent change in FRS over 10 years. Inspection of the data showed that FRS change over time was not linear; we therefore included a quadratic term for time. Both the intercept and slope (time) were fitted as random effects. The main effect represents the mean FRS difference in 2007–2009 between the reference quartile (best performance) and other quartiles. The interaction term between quartiles of motor tests and time allows examining whether the association between FRS and motor function changed over time; non-linear differences in change were allowed by including interaction terms between the quadratic time term and quartiles of motor tests.

Lower SES, weight, and height are strongly associated with motor performances and FRS [21]. Poorer cognitive function is also associated with higher FRS [13] and worse motor function [22,23]. We examined whether our findings were explained by confounding (SES, weight, height) or mediated by cognition by including the following covariates in models as main effects together with their significant (p < 0.05) interactions with time: SES (high vs intermediate/low), quartiles of BMI and height, the measure of cognition (AH4-I). These analyses used time invariant covariates defined in 2007–2009, and were replicated using time-dependent covariates; they were adjusted for age in 2007–2009 and sex and their interactions with time (linear, squared).

In sensitivity analyses, we examined the influence of incident mobility limitations before the assessment of motor function by excluding participants who reported mobility limitations (limited to climb several flights of stairs or to walk more than 1 mile) at least once between 1991–1993 and 2002–2004.

As FRS is higher in men than women [13], and men perform better than women on motor tests [20], we examined whether sex modified cross-sectional associations between the FRS and motor tests. We also investigated whether age modified their association.

Two-tailed p-values ≤ 0.05 were considered to be statistically significant. Statistical analyses were performed using SAS 9.2 (SAS Institute, Cary, North Carolina, USA).

## 3 Results

Of 10,308 participants of Whitehall II at inception (1985–1988), 8104 participated in the clinical 1991–1993 examination, 954 died before 2007, and 6225 participated in the 2007–2009 clinical examination; compared to those who participated in 2007–2009, those who did not were older (p < 0.001), more often women (p < 0.001), and had higher FRS (p < 0.001) in 1991–1993. We excluded 725 participants with a history of CHD/stroke, and 124 participants who did not have FRS or motor data. Our analyses are based on 5376 participants. The chair rise test was missing for 7.2% of the participants due to more stringent exclusion criteria than for other tests.

Participants' characteristics at four waves are shown in Table 1; 3250 (60%) participants had four FRS measurements, 1225 (23%) three, 633 (12%) two, and 268 (5%) one. Mean (SD) FRS increased from 8.6% (6.3) in 1991–1993 to 16.9% (10.6) in 2007–2009 (on average 16.8 years later) with the prevalence of vascular risk factors, besides smoking, also rising over this period. Higher age, male sex, and vascular risk factors were strongly associated with higher cardiovascular risk (Table 2); after adjustment for age and sex, higher BMI, smaller height, lower SES, and worse cognitive function remained associated with higher risk. Participants who developed mobility limitations between 1991–1993 and 2002–2004 had higher FRS than those who did not.

**Fig. 1:**
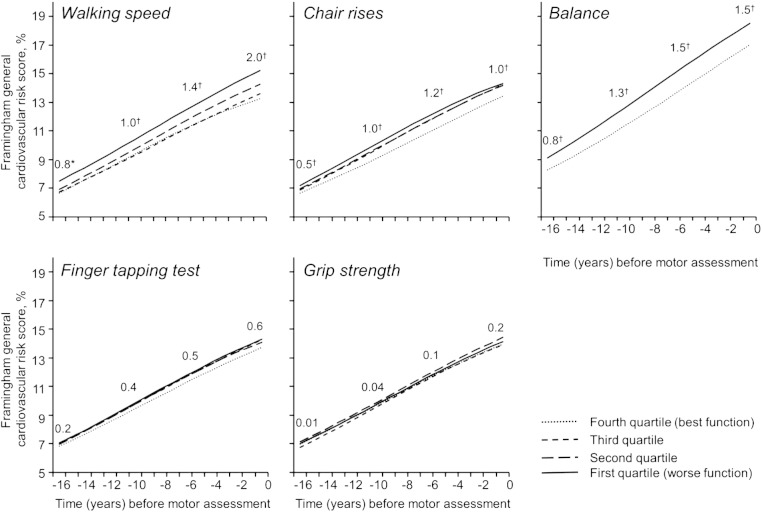
Predicted trajectories of the Framingham general cardiovascular risk score from 1991–1993 to 2007–2009 by quartiles of motor tests assessed in 2007–2009. Graphs are based on the back transformation of the log-FRS predicted by linear mixed models (Table 4). Quartiles of walking speed, chair rises, finger tapping, and grip strength were defined based on age- and sex-specific distributions (supplementary Table 2): Q4 (best function), dotted line; Q3, short-dashed line; Q2, long-dashed line; Q1 (worst function), solid line. For balance (c): passed, dotted line; failed, solid line; this graph corresponds to men aged 65 years in 2007–2009. Numbers on top of curves represent FRS differences between Q4 and Q1 (or between those who passed and failed the balance test) 16, 10, 5, and 0 years before motor function was assessed (*p < 0.05, † p < 10^− 3^).

Geometric FRS means from 1991–1993 to 2007–2009 according to quartiles of motor tests are presented in Table 3. Differences between the top and bottom quartiles were larger for walking speed, chair rises, and balance than for grip strength and finger tapping, and these differences increased somewhat over time. The results of modeling FRS trajectories are presented in Table 4 and Fig. 1. There was good agreement between observed (Table 3) and predicted differences (Fig. 1) in FRS between top and bottom quartiles of motor tests. For walking speed, chair rises, and balance, participants with poor performances in 2007–2009 had higher FRS concurrently as well as in 1991–1993, on average 16 years earlier. The largest differences were observed for walking speed, followed by balance, and finally chair rises. After back-transformation of the log-FRS, differences in FRS across groups tended to increase over time and be larger in 2007–2009 than in 1991–1993 (Fig. 1). FRS was not associated with measures taken at upper limbs (grip strength, finger tapping) at any time point.

Supplementary Tables 3–7 describe associations between participants' characteristics, including FRS components, and motor tests. Associations between FRS and walking speed, chair rises, and balance were explained by a single covariate; note that the weaker association between low total cholesterol and slow walking speed disappeared (p = 0.19) after adjustment for HDL-cholesterol. Finger tapping and grip strength were not associated with most FRS components.

Adjustment for cognitive status in 2007–2009 had little influence (supplementary Fig. 1, panel a). After adjustment for SES, BMI, and height, the association between FRS and motor tests decreased but remained statistically significant, except for balance in 2007–2009 (supplementary Fig. 1, panel b); analyses using time-dependent covariates yielded similar conclusions (data not shown). After exclusion of participants who reported mobility limitations, associations remained statistically significant in 1991–1993 for walking speed and balance (supplementary Fig. 1, panel c). There were no sex- (p-values > 0.10) or age-related differences (p-values > 0.10) in cross-sectional associations between motor tests and FRS.

## 4 Discussion

Higher Framingham cardiovascular risk was associated with worse motor function assessed through measures of walking speed, chair rises, and balance. Based on repeated FRS assessments, higher risk was associated with worse motor function 16 years later. This association was robust to adjustments for BMI, height, SES, and cognitive function; it was not explained by mobility limitations before motor assessment. Our findings suggest that exposure to vascular risk factors in midlife is associated with poor motor function at older ages. The corollary, that low vascular risk in midlife may delay loss of motor function and extend independent living in the elderly, could become a key clinical and public health perspective across rapidly aging societies.

It is well recognized that cerebral multi-infarct states can lead to gait disorders, but the contribution of vascular risk factors to age-related changes in motor function has received little research attention. Our findings show that high vascular risk is associated with poorer motor function. This is in agreement with previous studies, mainly based on walking speed, that reported associations of worse motor function with individual vascular risk factors (hypertension [5,24], diabetes [6], homocysteine [25–27], low HDL-cholesterol [28]) or markers of vascular aging (increased common carotid artery intima–media thickness [7,8], arterial stiffness [9]). Most prior studies were cross-sectional or based on a single measure of vascular risk, while we assessed cardiovascular risk four times over many years, thus minimizing the risk of measurement error and reverse causation. To our knowledge, our study is the first investigation of FRS in relation to motor function. This score is widely used in clinical practice and has the advantage of integrating several risk factors.

Several mechanisms may account for the association between vascular risk factors and poor motor function. First, vascular risk factors are associated with an increased risk of cardio- and cerebrovascular events which lead to poor motor function. We excluded participants with overt vascular disease and our findings cannot be explained by such events. It is possible, however, that vascular health contributes to this association given the link between vascular outcomes such as peripheral artery disease [29] and heart failure [30] and physical functioning. Second, vascular risk factors are associated with an increased risk of white matter lesions, a marker of vascular brain injury assessed through brain MRI. Higher volumes of white matter lesions, particularly in the periventricular region, are associated with poor motor function, probably by disrupting brain circuits involved in motor control [31,32], and represent a ‘central’ vascular component. Third, vascular risk factors are associated with worse cognitive performances [1], which are themselves associated with motor function [22,23]. However, our results remained unchanged in analyses adjusted for a cognitive test associated with FRS in our study [13]; the association between cardiovascular risk and motor function was not explained by cognitive status.

There was some heterogeneity across motor tests. The association with FRS was strongest for walking speed and balance, less pronounced for chair rises, and absent for finger tapping and grip strength. These differences likely reflect the fact that walking speed, balance, and chair rises represent general measures of motor function, since they involve several systems and functions, including cardiovascular function, while finger tapping and grip strength pertain to more specific functions (psychomotor speed, muscle function). Another potential explanation is that vascular risk factors increase the risk of peripheral neuropathy in diabetic patients [33], which could explain the apparent sparing of upper limbs; however, analyses excluding diabetic patients yielded similar conclusions.

The absolute difference in cardiovascular risk between quartiles of motor function is modest. For instance, for walking speed, the absolute difference in the 2007–2009 FRS between the worse and best performers was 2.0%. However, the difference in walking speed between the top and bottom quartiles is large (~ 0.6–0.7 m/s). A meta-analysis estimated that 0.1 m/s higher walking speed was associated with a 12% reduced risk of mortality [4]; therefore, a 0.6 m/s difference corresponds to a roughly 50% reduced risk of death and is clinically relevant as it would significantly impact cardiovascular mortality [34,35].

Previous studies that examined a vascular contribution to motor performances were performed in elderly subjects. Selection biases related to competing risks of death as well as reverse causation may complicate their interpretation. Our analyses, in contrast, are based on subjects who were on average ~ 49 years old at baseline followed for approximately 16 years; one important finding is that differences in FRS across groups defined by motor tests were already established many years before the motor assessment and well before the start of old age. However, the main limitation of our analyses is that motor function was measured once, and we were not able to study the association between the FRS and change in motor function. This limits causal inference as we were not able to formally assess temporality and to examine when differences in motor function appeared among participants; results of analyses with exclusion of persons who developed mobility limitations are reassuring to this respect. Our analyses are based on participants who participated in 2007–2009, which raises that possibility that selection biases due to survival or other causes may impact our findings. This is however unlikely given that the association between the FRS and motor tests was not modified by age; because persons who did not participate in 2007–2009 had higher FRS in 1991–1993 and are likely to be in worse health and have worse motor function [36], our estimates of the association between motor function and FRS are likely to be conservative. Finally, Whitehall II participants are office-based civil servants and not fully representative of the British population, which may limit the generalizability of our findings; this is likely, however, to mainly affect the distribution of risk rather than the strength of associations.

Our study has several strengths, including its large size and extended follow-up with at least three FRS measures available for most participants, thus allowing modeling trajectories over time. The availability of tests assessing different aspects of motor function is also an important feature.

In conclusion, our findings are in line with research on cognitive function and dementia showing that higher vascular risk in midlife is associated with worse outcomes later in life [2]. Our study is the first to show that participants with poor motor function have higher cardiovascular risk and that this association was already present approximately 16 years before the assessment of motor function. These findings suggest that vascular risk factors play an important and under-recognized role in motor function, independently of their impact on cognition. Thus, better control of vascular risk factors in midlife may prevent physical impairment and disability in the elderly.

## Funding

ASM is supported by a “European Young Investigator Award” from the European Science Foundation. EJB and MS are supported by the British Heart Foundation. MK is supported by the BUPA Foundation, UK, the Academy of Finland, Finland, and an ESRC professorship. The Whitehall II study has been supported by grants from the British Medical Research Council (MRC); the British Heart Foundation; the British Health and Safety Executive; the British Department of Health; the National Heart, Lung, and Blood Institute (R01HL036310); and the National Institute on Aging (R01AG013196 and R01AG034454). The funders had no role in the design or conduct of the study, in the collection, analysis and interpretation of data, in the preparation or approval of the manuscript, and in the decision to submit it to publication.

## Figures and Tables

**Table 1:** Clinical characteristics of Whitehall II participants.

Characteristics	Year			
	1991–1993	1997–1999	2002–2004	2007–2009
	N = 4699^a^	N = 3954^a^	N = 4427^a^	N = 5129^a^
Women, n (%)	1375 (29.3)	1104 (27.9)	1280 (28.9)	1475 (28.8)
Mean age (SD)	48.6 (5.8)	55.0 (5.8)	60.5 (5.8)	65.4 (5.8)
High grade, n (%)	2316 (49.3)	2011 (50.9)	2201 (49.7)	2527 (49.3)
Mean BMI (SD), kg/m^2^	25.0 (3.5)	25.8 (3.8)	26.5 (4.2)	26.6 (4.4)
Mean height (SD), cm	173 (9)	173 (9)	171 (9)	171 (9)
Mean SBP (SD), mm Hg	119 (13)	123 (16)	127 (16)	126 (16)
Mean DBP (SD), mm Hg	79 (9)	78 (10)	74 (10)	71 (10)
Antihypertensive drugs, n (%)	201 (4.3)	372 (9.4)	838 (18.9)	1576 (30.7)
Mean HDL cholesterol (SD), mmol/L	1.45 (0.41)	1.47 (0.39)	1.59 (0.44)	1.61 (0.45)
Mean total cholesterol (SD), mmol/L	6.38 (1.13)	5.92 (1.05)	5.78 (0.99)	5.30 (1.06)
Current smokers, n (%)	478 (10.2)	299 (7.6)	299 (6.7)	361 (7.0)
Diabetes, n (%)	95 (2.0)	155 (3.9)	313 (7.1)	564 (11.0)
Mean cognitive score (AH4-I) (SD)^b^	47 (10)	48 (10)	45 (11)	44 (11)
No mobility limitation^c^	3410 (72.6)	2652 (68.5)	2866 (65.6)	3069 (60.3)
Mean (geometric) FRS (SD), %	8.6 (6.3)	11.5 (7.9)	15.0 (9.7)	16.9 (10.6)
Mean walking speed (SD), m/s	–	–	–	1.11 (0.27)^d^
Mean time for five chair rises (SD), s	–	–	–	10.9 (3.3)^d^
Failed balance test, n (%)	–	–	–	2238 (44.5)^d^
Mean number of taps per 10 s (finger tapping test) (SD)	–	–	–	54.0 (10.8)^d^
Mean grip strength (SD), kg	–	–	–	36.2 (10.4)^d^

BMI, body mass index; SBP, systolic blood pressure; DBP, diastolic blood pressure; FRS, Framingham cardiovascular disease.

a Number of participants with the FRS at each examination.

b 1991–1993, N = 2100; 1997–1999, N = 3663; 2002–2004, N = 4357; 2007–2009, N = 5036.

c No difficulty to climb several flights of stairs and to walk more than 1 mile.

d 5376 participants had at least one motor test in 2007–2009 and at least one FRS during the follow-up. Missing values for motor tests: walking speed, N = 31; grip strength, N = 56; FTT, N = 16; chair rises, N = 387; balance, N = 95.

**Table 2:** Correlates of the Framingham general cardiovascular risk score assessed in 2007–2009.

Characteristics	Framingham general cardiovascular risk score				
	< 10%	10–14.9%	15–19.9%	≥ 20%	p^a^
	N = 1446	N = 1163	N = 969	N = 1551	
Women, % (95% CI)	74 (72–76)	25 (23–28)	10 (8–12)	6 (5–7)	< 10^− 3^
Mean age (SE)	62.2 (0.1)	65.5 (0.2)	67.2 (0.2)	69.7 (0.2)	< 10^− 3^
High grade, % (95% CI)	51 (46–57)	50 (47–53)	49 (45–53)	41 (38–43)	< 10^− 3^
Mean BMI (SE), kg/m^2^	24.7 (0.1)	26.9 (0.1)	28.0 (0.1)	29.1 (0.1)	< 10^− 3^
Mean height (SE), cm	168.7 (0.2)	167.8 (0.2)	168.3 (0.2)	167.3 (0.2)	< 10^− 3^
Mean SBP (SE), mm Hg	112.4 (0.4)	125.4 (0.4)	132.0 (0.5)	140.4 (0.4)	< 10^− 3^
Mean DBP (SE), mm Hg	64.4 (0.3)	71.2 (0.3)	75.1 (0.3)	78.5 (0.3)	< 10^− 3^
Antihypertensive drugs, % (95% CI)	12 (9–16)	25 (23–28)	37 (33–41)	50 (46–55)	< 10^− 3^
Mean HDL cholesterol (SE), mmol/L	1.93 (0.01)	1.67 (0.01)	1.57 (0.01)	1.38 (0.01)	< 10^− 3^
Mean total cholesterol (SE), mmol/L	5.00 (0.03)	5.40 (0.03)	5.66 (0.04)	5.86 (0.03)	< 10^− 3^
Current smokers, % (95% CI)	0.7 (0.4–1.0)	4 (2–5)	9 (5–12)	20 (16–25)	< 10^− 3^
Diabetes, % (95% CI)	1.3 (0.8–1.7)	6 (4–7)	12 (9–16)	31 (27–36)	< 10^− 3^
Mean cognitive score (AH4-I) (SE)	43.7 (0.3)	42.0 (0.3)	41.9 (0.4)	41.0 (0.3)	< 10^− 3^
No mobility limitation, % (95% CI)^b^	54 (48–60)	49 (46–52)	45 (42–49)	41 (36–45)	< 10^− 3^

BMI, body mass index; SBP, systolic blood pressure; DBP, diastolic blood pressure.

a p-Values computed using linear regression or analysis of covariance adjusted for age and sex with the log-transformed FRS as the dependent variable and participants' characteristics as explanatory variables. Age- and sex-adjusted means (SE) and percentages (95% CI) are presented.

b Defined as no self-reported difficulty to climb several flights of stairs and to walk more than 1 mile before the motor assessment according to questionnaires from 1991–1993, 1997–1999, and 2002–2004.

**Table 3:** Geometric mean of the Framingham general cardiovascular risk score (1991–1993, 1997–1999, 2002–2004, 2007–2009) by categories of motor function, assessed in 2007–9.

Test of motor function	Age- and sex-specific quartile	Framingham general cardiovascular risk score (%)							
		1991–1993		1997–1999		2002–2004		2007–2009	
		Geo. mean	95% CI	Geo. mean	95% CI	Geo. mean	95% CI	Geo. mean	95% CI
Walking speed (m/s)	Q4	6.5	6.3; 6.8	9.0	8.6; 9.3	11.8	11.4; 12.3	13.6	12.7; 13.6
	Q3	6.6	6.4; 6.9	9.1	8.7; 9.4	11.8	11.3; 12.3	14.0	13.0; 14.0
	Q2	6.8	6.6; 7.1	9.2	8.8; 9.6	12.5	12.0; 13.0	14.6	13.6; 14.6
	Q1	7.2	6.9; 7.5	9.8	9.4; 10.2	13.1	12.6; 13.6	15.6	14.5; 15.6
	Difference (Q1–Q4)	0.7	–	0.8	–	1.3	–	2.0	–
Five chair rises (time in seconds for 5 chair rises)	Q4	6.5	6.2; 6.8	8.7	8.4; 9.1	11.6	11.1; 12.0	13.9	12.9; 13.9
	Q3	6.7	6.4; 7.0	9.2	8.8; 9.6	12.3	11.8; 12.8	14.6	13.6; 14.6
	Q2	6.8	6.6; 7.1	9.2	8.8; 9.6	12.4	11.9; 12.9	14.5	13.5; 14.5
	Q1	7.1	6.8; 7.4	9.5	9.1; 10.0	12.7	12.2; 13.2	14.7	13.6; 14.7
	Difference (Q1–Q4)	0.6	–	0.8	–	1.1	–	0.8	–
Balance test	Passed	6.1	6.0; 6.3	8.4	8.2; 8.6	11.2	10.9; 11.5	13.5	12.9; 13.5
	Failed	7.7	7.5; 7.9	10.4	10.0; 10.7	13.7	13.3; 14.1	15.3	14.5; 15.3
	Difference (failed–passed)	1.6	–	2.0	–	2.5	–	1.8	–
Finger tapping test (number of taps per 10 s)	Q4	6.7	6.5; 7.0	9.1	8.7; 9.5	12.1	11.6; 12.5	14.1	13.1; 14.1
	Q3	6.8	6.6; 7.1	9.4	9.0; 9.8	12.4	11.9; 12.8	14.6	13.6; 14.6
	Q2	6.8	6.6; 7.1	9.1	8.7; 9.5	12.3	11.9; 12.8	14.4	13.4; 14.4
	Q1	6.8	6.6; 7.1	9.4	9.0; 9.8	12.4	11.9; 12.9	14.7	13.7; 14.7
	Difference (Q1–Q4)	0.1	–	0.3	–	0.4	–	0.7	–
Grip strength (kg)	Q4	6.8	6.6; 7.1	9.4	9.0; 9.7	12.3	11.9; 12.8	14.3	13.4; 14.3
	Q3	6.6	6.3; 6.9	9.1	8.7; 9.5	12.3	11.8; 12.8	14.2	13.2; 14.2
	Q2	7.0	6.7; 7.3	9.3	8.9; 9.8	12.5	12.0; 13.0	14.8	13.8; 14.8
	Q1	6.8	6.5; 7.1	9.1	8.7; 9.6	12.1	11.6; 12.6	14.5	13.5; 14.5
	Difference (Q1–Q4)	0.0	–	− 0.2	–	− 0.2	–	0.2	–

**Table 4:** Trajectories of the log-transformed Framingham general cardiovascular risk score (FRS) from 1991–1993 to 2007–2009 according to quartiles of tests of motor function (MT) assessed in 2007–2009.

Parameter	Walking speed (N = 5336) (m/s)			Five chair rises (N = 4958) (time in seconds)			Balance test (N = 5268) (failed vs passed)			Finger tapping test (N = 5348) (number of taps per 10 s)			Grip strength (N = 5319) (kg)		
	Beta	95% CI	p^b^	Beta	95% CI	p^b^	Beta^c^	95% CI	p	Beta	95% CI	p^b^	Beta	95% CI	p^b^
Intercept	− 2.022	− 2.056; − 1.987	< 10^− 4^	− 2.007	− 2.043; − 1.971	< 10^− 4^	− 1.769	− 1.788; − 1.751	< 10^− 4^	− 1.986	− 2.020; − 1.952	< 10^− 4^	− 1.969	− 2.003; − 1.935	< 10^− 4^
MT-Q4^a^	0.000	(Reference)		0.000	(Reference)		0.000	(Reference)		0.000	(Reference)		0.000	(Reference)	
MT-Q3	0.027	− 0.022; 0.076		0.056	0.006; 0.107		0.083	0.056; 0.110	< 10^− 4^	0.037	− 0.012; 0.086		− 0.005	− 0.054; 0.044	
MT-Q2	0.074	0.025; 0.123		0.054	0.003; 0.105					0.025	− 0.023; 0.074		0.032	− 0.016; 0.081	
MT-Q1	0.139	0.090; 0.188	< 10^− 4^	0.062	0.011; 0.113	0.013				0.040	− 0.01; 0.089	0.16	0.013	− 0.037; 0.063	0.38
Time	0.200	0.153; 0.247	< 10^− 4^	0.316	0.267; 0.365	< 10^− 4^	0.344	0.310; 0.378	< 10^− 4^	0.268	0.221; 0.315	< 10^− 4^	0.254	0.208; 0.300	< 10^− 4^
Time^2^	− 0.143	− 0.169; − 0.117	< 10^− 4^	− 0.077	− 0.104; − 0.050	< 10^− 4^	− 0.067	− 0.086; − 0.049	< 10^− 4^	− 0.106	− 0.131; − 0.08	< 10^− 4^	− 0.112	− 0.137; − 0.086	< 10^− 4^
Time × MT-Q4	0.000	(Reference)		0.000	(Reference)		0.000	(Reference)		0.000	(Reference)		0.000	(Reference)	
Time × MT-Q3	0.081	0.014; 0.148		− 0.054	− 0.124; 0.015		− 0.047	− 0.098; 0.003		0.004	− 0.063; 0.072		− 0.013	− 0.079; 0.054	
Time × MT-Q2	0.066	− 0.002; 0.133		− 0.056	− 0.126; 0.014					− 0.032	− 0.099; 0.035		0.024	− 0.043; 0.091	
Time × MT-Q1	0.076	0.008; 0.144		− 0.103	− 0.172; − 0.033					− 0.015	− 0.082; 0.053		0.010	− 0.058; 0.079	
Time^2^ × MT-Q4	0.000	(Reference)		0.000	(Reference)		0.000	(Reference)		0.000	(Reference)		0.000	(Reference)	
Time^2^ × MT-Q3	0.045	0.008; 0.081		− 0.043	− 0.081; − 0.005		− 0.024	− 0.052; 0.004	0.18	0.001	− 0.036; 0.037		− 0.020	− 0.057; 0.016	
Time^2^ × MT-Q2	0.027	− 0.009; 0.064		− 0.038	− 0.076; 0.000					− 0.022	− 0.059; 0.014		0.012	− 0.025; 0.048	
Time^2^ × MT-Q1	0.039	0.001; 0.076	0.095	− 0.058	− 0.096; − 0.020	0.020				− 0.013	− 0.050; 0.024	0.47	0.002	− 0.035; 0.039	0.85

Trajectories of the log-transformed FRS were modeled using linear mixed models with random effects for the intercept and time (linear, squared) and a backwards time scale. The main effects of the quartiles of the motor tests represent the difference in the log-transformed FRS between each quartile and the reference quartile in 2007–2009, and the interactions of the quartiles of the motor tests with time and time squared examine whether trajectories of the FRS are different in each quartile compared to the reference quartile. Regression coefficients (beta) were back transformed to present these findings graphically (Fig. 1).

a MT-Q1 to -Q4: age- and sex-specific quartiles of motor tests from worse (Q1) to better function (Q4, reference); for the balance test, Q4 corresponds to those who passed the test and Q3 to those who failed the test.

b p-Values for trend for the main effect of the quartiles of the motor tests and for their interactions with time (linear, squared) computed by including a four-level ordinal variable defined by the mean of the tests in each of the quartiles. We report a global test for the interactions of motor tests with time (linear, squared).

c The linear mixed model was adjusted for age (centered at 65 years; p < 10^− 4^) and sex (reference group, males; p < 10^− 4^), the interactions of age and sex with time and time squared (all p < 10^− 4^), the interaction between age and sex (p < 10^− 4^), and the three-way interaction between age, sex, and time (p = 0.002).
