# Supplementary material for: Trajectories of the Framingham general cardiovascular risk profile in midlife and poor motor function later in life: The Whitehall II study
Source: Int J Cardiol. 2014 Mar 1;172(1):96–102. doi: 10.1016/j.ijcard.2013.12.051 (PMC3991855; doi:10.1016/j.ijcard.2013.12.051)

SUPPLEMENTAL MATERIAL

Supplementary table 1 **Correlations between motor tests**

Standardized tests of motor function	Age- and sex-adjusted partial Spearman correlation coefficients*			Beta (SE)†
	Time for 5 chair rises	Finger tapping test	Grip strength	Failed balance test
Walking speed (m/s)	-0.16	0.06	0.21	-0.28 (0.03)
Time for 5 chair rises (seconds)	--	-0.25	-0.21	0.37 (0.03)
Finger tapping test (number of taps/10 seconds)	--	--	0.25	-0.21 (0.03)
Grip strength (kg)	--	--	--	-0.22 (0.02)

Analyses are based on z-scores of walking speed, time for 5 chair rises, finger tapping test, and grip strength.
* All $p<10^{-4}$.
† Beta regression coefficients and standard errors from separate analyses of covariance adjusted for age and sex with the balance test as the independent variable and other tests as dependent variables; all $p<10^{-4}$.

Supplementary table 2 **Definition of age- and sex-specific quartiles of motor tests**

Sex	Age (y)	Cutoffs Q1	Q2	Q3	Q4	Average value (SD) in each quartile Q1	Q2	Q3	Q4	Δ*
**Walking speed (m/s)**										
Men	<61	<1.01	1.01-1.16	1.17-1.34	>1.34	0.87 (0.12)	1.09 (0.04)	1.25 (0.05)	1.51 (0.15)	0.64
Men	61-64	<1.00	1.00-1.15	1.16-1.34	>1.34	0.86 (0.12)	1.08 (0.04)	1.25 (0.06)	1.50 (0.15)	0.64
Men	65-70	<0.96	0.96-1.12	1.13-1.31	>1.31	0.81 (0.12)	1.05 (0.05)	1.22 (0.05)	1.49 (0.14)	0.68
Men	>70	<0.87	0.87-1.04	1.05-1.22	>1.22	0.72 (0.12)	0.96 (0.05)	1.13 (0.05)	1.39 (0.16)	0.66
Women	<61	<0.92	0.92-1.09	1.10-1.24	>1.24	0.79 (0.11)	1.00 (0.05)	1.15 (0.05)	1.41 (0.15)	0.62
Women	61-64	<0.89	0.89-1.04	1.05-1.20	>1.20	0.75 (0.12)	0.97 (0.05)	1.12 (0.05)	1.36 (0.14)	0.61
Women	65-70	<0.84	0.84-0.97	0.98-1.13	>1.13	0.67 (0.15)	0.91 (0.04)	1.05 (0.04)	1.28 (0.13)	0.62
Women	>70	<0.71	0.71-0.87	0.88-1.03	>1.03	0.56 (0.12)	0.79 (0.05)	0.95 (0.04)	1.20 (0.15)	0.64
**Time (seconds) for 5 chair rises**										
Men	<61	>11.9	10.0-11.9	8.3-9.9	<8.3	14.5 (3.0)	10.9 (0.6)	9.1 (0.5)	7.2 (0.9)	-7.3
Men	61-64	>12.0	10.0-12.0	8.3-9.9	<8.3	14.3 (2.4)	10.8 (0.6)	9.1 (0.4)	7.3 (0.8)	-7.0
Men	65-70	>12.3	10.5-12.3	8.9-10.4	<8.9	14.9 (3.0)	11.3 (0.5)	9.6 (0.4)	7.7 (0.9)	-7.1
Men	>70	>13.3	11.1-13.3	9.3-11.0	<9.3	16.2 (3.3)	12.0 (0.6)	10.2 (0.5)	8.2 (0.9)	-8.0
Women	<61	>12.2	10.3-12.2	8.5-10.2	<8.5	14.9 (4.4)	11.1 (0.6)	9.4 (0.5)	7.4 (0.9)	-7.6
Women	61-64	>12.4	10.6-12.4	8.9-10.5	<8.9	15.3 (3.0)	11.3 (0.6)	9.7 (0.5)	7.8 (0.8)	-7.5
Women	65-70	>13.3	11.1-13.3	9.3-11.0	<9.3	16.9 (3.8)	12.1 (0.7)	10.2 (0.5)	8.1 (0.9)	-8.8
Women	>70	>14.5	11.9-14.5	9.7-11.8	<9.7	18.0 (4.5)	13.1 (0.7)	10.8 (0.6)	8.5 (1.0)	-9.5
**Finger tapping test (number of taps per 10 seconds)**										
Men	<61	<51.0	51.0-57.7	57.8-64.7	>64.7	45.1 (5.6)	54.2 (1.9)	60.9 (2.0)	70.3 (4.9)	25.1
Men	61-64	<50.7	50.7-57.7	57.8-64.7	>64.7	43.3 (6.3)	54.0 (2.0)	60.8 (2.0)	70.3 (4.5)	26.9
Men	65-70	<48.7	48.7-55.0	55.1-61.0	>61.0	42.2 (5.8)	51.7 (1.8)	57.6 (1.6)	67.1 (4.7)	24.9
Men	>70	<44.7	44.7-51.7	51.8-59.3	>59.3	38.3 (5.6)	48.0 (2.0)	55.2 (2.1)	65.1 (4.5)	26.8
Women	<61	<46.7	46.7-53.3	53.4-59.7	>59.7	40.8 (4.7)	50.0 (1.9)	56.0 (1.7)	64.8 (4.3)	24.0
Women	61-64	<43.3	43.3-51.3	51.4-58.0	>58.0	37.0 (6.0)	47.5 (2.2)	54.6 (2.0)	63.8 (4.4)	26.8
Women	65-70	<41.0	41.0-48.3	48.4-54.7	>54.7	33.4 (7.3)	44.4 (2.0)	51.5 (1.8)	59.6 (4.0)	26.2
Women	>70	<39.0	39.0-46.0	46.1-52.7	>52.7	32.0 (5.7)	42.5 (2.0)	48.7 (1.9)	57.6 (4.1)	25.7
**Grip strength (kg)**										
Men	<61	<37.0	37.0-42.7	42.8-48.0	>48.0	31.2 (4.8)	39.8 (1.6)	45.1 (1.5)	53 (4.3)	21.8
Men	61-64	<37.0	37.0-42.0	42.1-47.3	>47.3	31.3 (4.6)	39.5 (1.4)	44.4 (1.6)	51.5 (3.4)	20.2
Men	65-70	<35.0	35.0-40.3	40.4-45.3	>45.3	29.7 (4.2)	37.7 (1.6)	42.6 (1.5)	49.5 (3.5)	19.8
Men	>70	<30.3	30.3-36.3	36.4-41.5	>41.5	25.4 (4.4)	33.2 (1.8)	38.7 (1.5)	46.1 (3.8)	20.7
Women	<61	<23.3	23.3-27.3	27.4-31.3	>31.3	19.3 (3.2)	25.3 (1.2)	29.0 (1.1)	33.9 (2.0)	14.6
Women	61-64	<22.3	22.3-26.0	26.1-29.7	>29.7	18.8 (3.2)	24.2 (1.0)	27.7 (1.0)	32.8 (2.8)	14.0
Women	65-70	<21.0	21.0-24.7	24.8-28.0	>28.0	16.4 (4.0)	22.7 (1.0)	26.3 (0.9)	31.3 (3.0)	14.9
Women	>70	<17.7	17.7-22.0	22.1-26.0	>26.0	14.0 (2.6)	19.9 (1.3)	23.8 (1.2)	28.7 (2.5)	14.7

For all tests, except for chair rises, higher values of the test represent better function; for chair rises, a longer time represents poorer function. For all tests, Q1 represents poorer function and Q4 represents better function.
* Difference between top and bottom quartile.

Supplementary table 3 **Correlates of walking speed (2007-2009)**

	Age- and sex-specific quartiles of walking speed (m/s)				
	**Q4**	**Q3**	**Q2**	**Q1**	
**Characteristics**	**N=1374**	**N=1339**	**N=1335**	**N=1288**	**P***
High grade, % (95% CI)	57 (54-59)	53 (51-56)	47 (44-50)	40 (37-42)	$<10^{-3}$
Mean BMI (SE), kg/m²	25.9 (0.1)	26.2 (0.1)	26.8 (0.1)	27.7 (0.1)	$<10^{-3}$
Mean height (SE), cm	171.8 (0.2)	171.5 (0.2)	170.8 (0.2)	170.0 (0.2)	$<10^{-3}$
Mean SBP (SE), mmHg	124.6 (0.4)	125.6 (0.4)	126.0 (0.5)	126.1 (0.4)	$<10^{-3}$
Mean DBP (SE), mmHg	71.0 (0.3)	71.4 (0.3)	71.6 (0.3)	71.6 (0.3)	0.013
Antihypertensive drugs, % (95% CI)	25 (23-28)	28 (26-31)	33 (31-36)	38 (35-40)	$<10^{-3}$
Mean HDL cholesterol (SE), mmol/l	1.66 (0.01)	1.62 (0.01)	1.61 (0.01)	1.56 (0.01)	$<10^{-3}$
Mean total cholesterol (SE), mmol/l	5.34 (0.03)	5.31 (0.03)	5.31 (0.04)	5.25 (0.03)	0.031
Mean triglycerides (SE), mmol/l	6 (5-8)	6 (4-7)	8 (6-9)	9 (7-10)	$<10^{-3}$
Current smokers, % (95% CI)	8 (7-10)	10 (9-12)	10 (9-12)	16 (14-19)	$<10^{-3}$
Diabetes, % (95% CI)	46.2 (0.3)	44.8 (0.3)	43.6 (0.4)	40.3 (0.3)	$<10^{-3}$
Mean cognitive score (AH4-I) (SE)	58 (55-61)	49 (47-52)	46 (43-49)	35 (33-38)	$<10^{-3}$
No mobility limitation, % (95% CI)†	57 (54-59)	53 (51-56)	47 (44-50)	40 (37-42)	$<10^{-3}$

BMI, body mass index; SBP, systolic blood pressure; DBP, diastolic blood pressure; Q, quartile (from best – Q4 – to worst – Q1).
* P-values were computed using linear regression or analysis of covariance adjusted for age and sex with walking speed as the dependent variable and participants' characteristics as explanatory variables. Age- and sex-adjusted means (SE) and percentages (95% CI) are presented.
† Defined as no self-reported difficulty to climb several flights of stairs and to walk more than one mile before the motor assessment according to questionnaires from 1991-1993, 1997-1999, and 2002-2004.

Supplementary table 4 **Correlates of grip strength (2007-2009)**

Characteristics	Age- and sex-specific quartiles of grip strength (kg)				
	**Q4**	**Q3**	**Q2**	**Q1**	
	**N=1407**	**N=1352**	**N=1317**	**N=1243**	**P***
High grade, % (95% CI)	55 (53-58)	51 (48-53)	48 (46-51)	43 (40-45)	$<10^{-3}$
Mean BMI (SE), kg/m²	27.0 (0.1)	26.6 (0.1)	26.5 (0.1)	26.5 (0.1)	$<10^{-3}$
Mean height (SE), cm	173.3 (0.2)	171.3 (0.2)	170.4 (0.2)	169.0 (0.2)	$<10^{-3}$
Mean SBP (SE), mmHg	126.2 (0.4)	125.8 (0.4)	125.5 (0.5)	125.0 (0.4)	0.025
Mean DBP (SE), mmHg	71.9 (0.3)	71.6 (0.3)	71.3 (0.3)	70.9 (0.3)	0.011
Antihypertensive drugs, % (95% CI)	31 (29-34)	29 (27-32)	32 (29-34)	32 (30-34)	0.26
Mean HDL cholesterol (SE), mmol/l	1.61 (0.01)	1.63 (0.01)	1.62 (0.01)	1.60 (0.01)	0.90
Mean total cholesterol (SE), mmol/l	5.36 (0.03)	5.32 (0.03)	5.30 (0.04)	5.23 (0.03)	$<10^{-3}$
Mean triglycerides (SE), mmol/l	1.26 (0.02)	1.23 (0.02)	1.26 (0.02)	1.26 (0.02)	0.75
Current smokers, % (95% CI)	6 (5-7)	7 (6-9)	7 (6-9)	7 (6-9)	0.15
Diabetes, % (95% CI)	8 (7-10)	10 (9-12)	12 (10-14)	14 (12-16)	$<10^{-3}$
Mean cognitive score (AH4-I) (SE)	44.7 (0.3)	44.5 (0.3)	43.6 (0.4)	42.3 (0.3)	$<10^{-3}$
No mobility limitation, % (95% CI)†	53 (51-56)	51 (48-54)	46 (43-49)	39 (36-42)	$<10^{-3}$

BMI, body mass index; SBP, systolic blood pressure; DBP, diastolic blood pressure; Q, quartile (from best – Q4 – to worst – Q1).
* P-values were computed using linear regression or analysis of covariance adjusted for age and sex with grip strength as the dependent variable and participants' characteristics as explanatory variables. Age- and sex-adjusted means (SE) and percentages (95% CI) are presented.
† Defined as no self-reported difficulty to climb several flights of stairs and to walk more than one mile before the motor assessment according to questionnaires from 1991-1993, 1997-1999, and 2002-2004.

Supplementary table 5 **Correlates of the finger tapping test (FTT) (2007-2009)**

	Age- and sex-specific quartiles of FTT (taps per 10 seconds)				
	**Q4**	**Q3**	**Q2**	**Q1**	
**Characteristics**	**N=1391**	**N=1323**	**N=1350**	**N=1284**	**P***
High grade, % (95% CI)	52 (50-55)	50 (47-52)	49 (47-52)	45 (43-48)	$<10^{-3}$
Mean BMI (SE), kg/m²	26.6 (0.1)	26.8 (0.1)	26.6 (0.1)	26.6 (0.1)	0.70
Mean height (SE), cm	171.5 (0.2)	171.1 (0.2)	170.9 (0.2)	170.5 (0.2)	$<10^{-3}$
Mean SBP (SE), mmHg	125.5 (0.4)	126.1 (0.4)	125.4 (0.5)	125.4 (0.4)	0.50
Mean DBP (SE), mmHg	71.6 (0.3)	71.5 (0.3)	71.2 (0.3)	71.3 (0.3)	0.65
Antihypertensive drugs, % (95% CI)	28 (26-31)	32 (29-34)	32 (30-35)	32 (30-35)	0.050
Mean HDL cholesterol (SE), mmol/l	1.62 (0.01)	1.61 (0.01)	1.62 (0.01)	1.61 (0.01)	0.25
Mean total cholesterol (SE), mmol/l	5.34 (0.03)	5.32 (0.03)	5.29 (0.04)	5.26 (0.03)	0.11
Mean triglycerides (SE), mmol/l	1.24 (0.02)	1.25 (0.02)	1.25 (0.02)	1.26 (0.02)	0.23
Current smokers, % (95% CI)	6 (5-7)	7 (5-8)	8 (6-9)	8 (6-9)	0.013
Diabetes, % (95% CI)	9 (8-11)	11 (10-13)	11 (9-13)	13 (11-15)	$<10^{-3}$
Mean cognitive score (AH4-I) (SE)	44.9 (0.3)	44.3 (0.3)	43.4 (0.4)	42.4 (0.3)	$<10^{-3}$
No mobility limitation, % (95% CI)†	49 (47-52)	49 (46-52)	47 (44-49)	44 (41-46)	$<10^{-3}$

BMI, body mass index; SBP, systolic blood pressure; DBP, diastolic blood pressure; Q, quartile (from best – Q4 – to worst – Q1).
* P-values were computed using linear regression or analysis of covariance adjusted for age and sex with the number of taps as the dependent variable and participants' characteristics as explanatory variables. Age- and sex-adjusted means (SE) and percentages (95% CI) are presented.
† Defined as no self-reported difficulty to climb several flights of stairs and to walk more than one mile before the motor assessment according to questionnaires from 1991-1993, 1997-1999, and 2002-2004.

Supplementary table 6 **Correlates of chair rises (2007-2009)**

	**Age- and sex-specific quartiles of chair rises (time in seconds per five chair rises)**				
	**Q1**	**Q2**	**Q3**	**Q4**	
**Characteristics**	**N=1255**	**N=1252**	**N=1282**	**N=1229**	**P***
High grade, % (95% CI)	54 (51-57)	51 (48-53)	50 (47-52)	46 (43-48)	$<10^{-3}$
Mean BMI (SE), kg/m²	25.7 (0.1)	26.6 (0.1)	26.6 (0.1)	27.1 (0.1)	$<10^{-3}$
Mean height (SE), cm	170.6 (0.2)	171.2 (0.2)	171.7 (0.2)	171.4 (0.2)	0.002
Mean SBP (SE), mmHg	125.5 (0.4)	126.4 (0.4)	125.5 (0.5)	124.8 (0.4)	0.23
Mean DBP (SE), mmHg	71.5 (0.3)	72.0 (0.3)	71.4 (0.3)	70.9 (0.3)	0.061
Antihypertensive drugs, % (95% CI)	26 (24-29)	29 (27-32)	30 (27-32)	35 (33-38)	$<10^{-3}$
Mean HDL cholesterol (SE), mmol/l	1.66 (0.01)	1.62 (0.01)	1.60 (0.01)	1.57 (0.01)	$<10^{-3}$
Mean total cholesterol (SE), mmol/l	5.35 (0.03)	5.34 (0.03)	5.34 (0.04)	5.20 (0.03)	$<10^{-3}$
Mean triglycerides (SE), mmol/l	1.17 (0.02)	1.25 (0.02)	1.27 (0.02)	1.30 (0.02)	$<10^{-3}$
Current smokers, % (95% CI)	5 (4-7)	8 (7-10)	7 (6-8)	7 (6-9)	0.38
Diabetes, % (95% CI)	10 (8-11)	10 (9-12)	10 (8-11)	13 (11-15)	$<10^{-3}$
Mean cognitive score (AH4-I) (SE)	45.1 (0.3)	44.2 (0.3)	43.8 (0.4)	43.1 (0.3)	$<10^{-3}$
No mobility limitation, % (95% CI)†	56 (53-59)	50 (47-52)	52 (49-55)	40 (37-43)	$<10^{-3}$

BMI, body mass index; SBP, systolic blood pressure; DBP, diastolic blood pressure; Q, quartile (from best – Q1 – to worst – Q4).

* P-values were computed using linear regression or analysis of covariance adjusted for age and sex with time to perform five chair rises as the dependent variable and participants' characteristics as explanatory variables. Age- and sex-adjusted means (SE) and percentages (95% CI) are presented.

† Defined as no self-reported difficulty to climb several flights of stairs and to walk more than one mile before the motor assessment according to questionnaires from 1991-1993, 1997-1999, and 2002-2004.

Supplementary table 7 **Correlates of balance (2007-2009)**

Characteristics	Balance test: Passed N=2905	Balance test: Failed N=2363	P*
Women, % (95% CI)	22 (20-24)	36 (34-38)	$<10^{-3}$
Mean age (SE)	63.7 (0.1)	67.4 (0.1)	$<10^{-3}$
High grade, % (95% CI)	52 (50-54)	46 (44-48)	$<10^{-3}$
Mean BMI (SE), kg/m²	25.8 (0.1)	27.7 (0.1)	$<10^{-3}$
Mean height (SE), cm	171.1 (0.1)	171.1 (0.1)	0.97
Mean SBP (SE), mmHg	125.5 (0.3)	125.6 (0.3)	0.84
Mean DBP (SE), mmHg	71.3 (0.2)	71.5 (0.2)	0.37
Antihypertensive drugs, % (95% CI)	27 (25-29)	36 (34-38)	$<10^{-3}$
Mean HDL cholesterol (SE), mmol/l	1.64 (0.01)	1.57 (0.01)	$<10^{-3}$
Mean total cholesterol (SE), mmol/l	5.37 (0.02)	5.22 (0.02)	$<10^{-3}$
Mean triglycerides (SE), mmol/l	1.19 (0.01)	1.31 (0.01)	$<10^{-3}$
Current smokers, % (95% CI)	6 (5-7)	9 (8-11)	$<10^{-3}$
Diabetes, % (95% CI)	8 (7-10)	14 (13-16)	$<10^{-3}$
Mean cognitive score (AH4-I) (SE)	44.8 (0.2)	42.6 (0.2)	$<10^{-3}$
No mobility limitation, % (95% CI)†	54 (52-56)	40 (38-42)	$<10^{-3}$

BMI, body mass index; SBP, systolic blood pressure; DBP, diastolic blood pressure.
* For participants' characteristics as continuous variables, P-values were computed using analysis of covariance adjusted for age and sex with balance as the independent variable; for participants' characteristics as binary variables, P-values were computed using logistic regression adjusted for age and sex with balance as the dependent variable. Age- and sex-adjusted means (SE) and percentages (95% CI) are presented.
† Defined as no self-reported difficulty to climb several flights of stairs and to walk more than one mile before the motor assessment according to questionnaires from 1991-1993, 1997-1999, and 2002-2004.

Supplementary figure 1 **Predicted trajectories of the Framingham general cardiovascular risk score (FRS) from 1991-1993 to 2007-2009 by tests of motor function (walking speed, chair rises, balance) assessed in 2007-2009: sensitivity analyses**

The graphs are based on the back transformation of the logarithm of the FRS predicted by the linear mixed models (all are adjusted for age centered at 65 years and sex with men as the reference, together with their interactions with time and time squared):
a) Adjustment for quartiles of AH4 (reference, highest quartile) in 2007-9;
b) Adjustment for quartiles of height (reference, highest quartile) and BMI (reference, lowest quartile) in 2007-9 and grade (reference, high grade);
c) Analyses restricted to participants who did not report mobility limitations between 1991-3 and 2007-9 (walking speed, N=2787; chair rises, N=2690; balance, N=2773).

For walking speed and chair rises, quartiles were defined based on their age- and sex-specific distributions (supplementary table 1): fourth quartile (best function), dotted line; third quartile, short-dashed line; second quartile, long-dashed line; first quartile (worst function), solid line. The graphs are drawn for participants in the reference category for adjustment variables. For the balance test: passed the test, dotted line; failed the test, solid line; this graph corresponds to men aged 65 years in 2007-9.

The numbers on top of the curves represent the difference between the fourth and first quartiles of the motor tests (or between those who passed or failed the balance test) 16, 10, 5, and 0 years before motor function was assessed (*p<0.05, †$p<10^{-3}$).

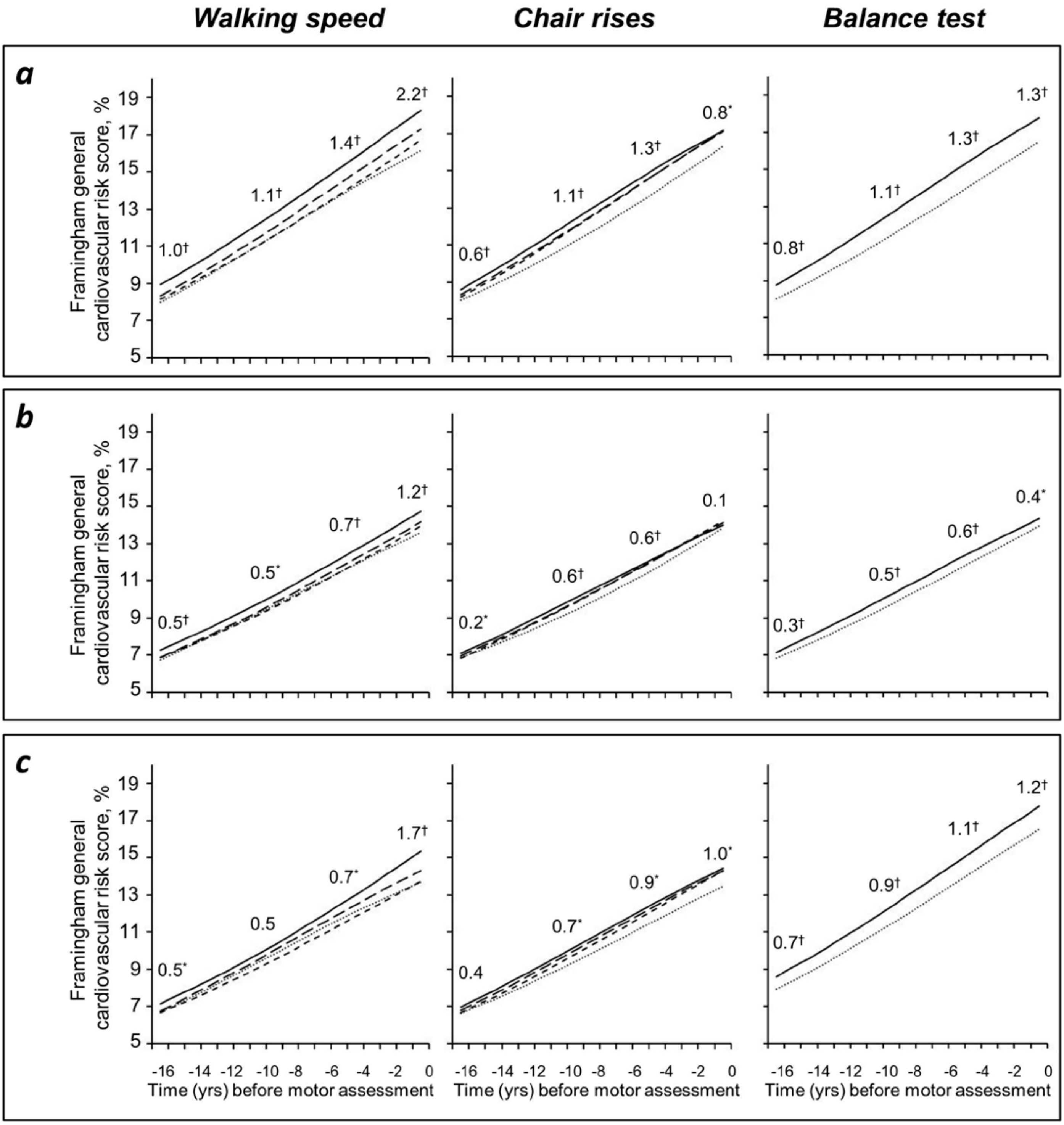

Supplement: Supplementary file 1 — Supplementary materials. [file mmc1.pdf]
